# Altering arabinans increases *Arabidopsis* guard cell flexibility and stomatal opening

**DOI:** 10.1016/j.cub.2022.05.042

**Published:** 2022-07-25

**Authors:** Sarah Carroll, Sam Amsbury, Clinton H. Durney, Richard S. Smith, Richard J. Morris, Julie E. Gray, Andrew J. Fleming

**Affiliations:** 1 School of Biosciences, University of Sheffield, Western Park, Sheffield S10 2TN, UK; 2 Computational and Systems Biology, John Innes Centre, Norwich Research Park, Norwich NR4 7UH, UK

**Keywords:** stomata, guard cell, mechanics, computational modelling, cell wall, Arabidopsis

## Abstract

Stomata regulate plant water use and photosynthesis by controlling leaf gas exchange. They do this by reversibly opening the pore formed by two adjacent guard cells, with the limits of this movement ultimately set by the mechanical properties of the guard cell walls and surrounding epidermis.^1^^,^^2^ A body of evidence demonstrates that the methylation status and cellular patterning of pectin wall polymers play a core role in setting the guard cell mechanical properties, with disruption of the system leading to poorer stomatal performance.^3^, ^4^, ^5^, ^6^ Here we present genetic and biochemical data showing that wall arabinans modulate guard cell flexibility and can be used to engineer stomata with improved performance. Specifically, we show that a short-chain linear arabinan epitope associated with the presence of rhamnogalacturonan I in the guard cell wall is required for full opening of the stomatal pore. Manipulations leading to the novel accumulation of longer-chain arabinan epitopes in guard cell walls led to an increase in the maximal pore aperture. Using computational modeling combined with atomic force microscopy, we show that this phenotype reflected a decrease in wall matrix stiffness and, consequently, increased flexing of the guard cells under turgor pressure, generating larger, rounder stomatal pores. Our results provide theoretical and experimental support for the conclusion that arabinan side chains of pectin modulate guard cell wall stiffness, setting the limits for cell flexing and, consequently, pore aperture, gas exchange, and photosynthetic assimilation.

## Highlights

• Cell walls in stomata have a distinct composition of arabinans
• Increasing the level of a specific arabinan makes the walls more flexible
• Stomata with more flexible walls can open wider
• Under high CO_2_, more flexible, wider stomata increase carbon assimilation rate

## Graphical abstract

**Figure:**
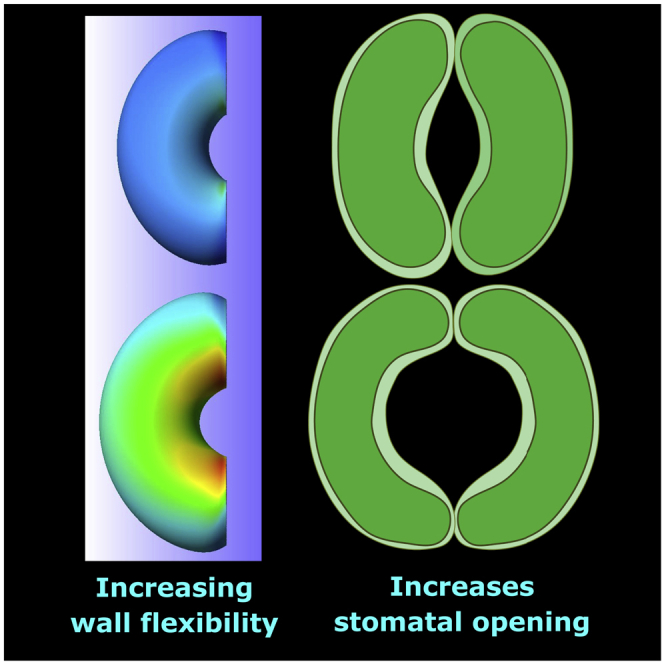

## Teaser

The degree of stomatal opening is set by the mechanical properties of the guard cell walls. Carroll et al. show that wall flexibility is set by the arabinan composition, and that by manipulating arabinan polymers it is possible to engineer stomata with increased opening under elevated CO_2_, leading to increased carbon assimilation.

## Results and discussion

### Guard cell walls are rich in short-chain linear arabinan epitopes

**Figure 1:**
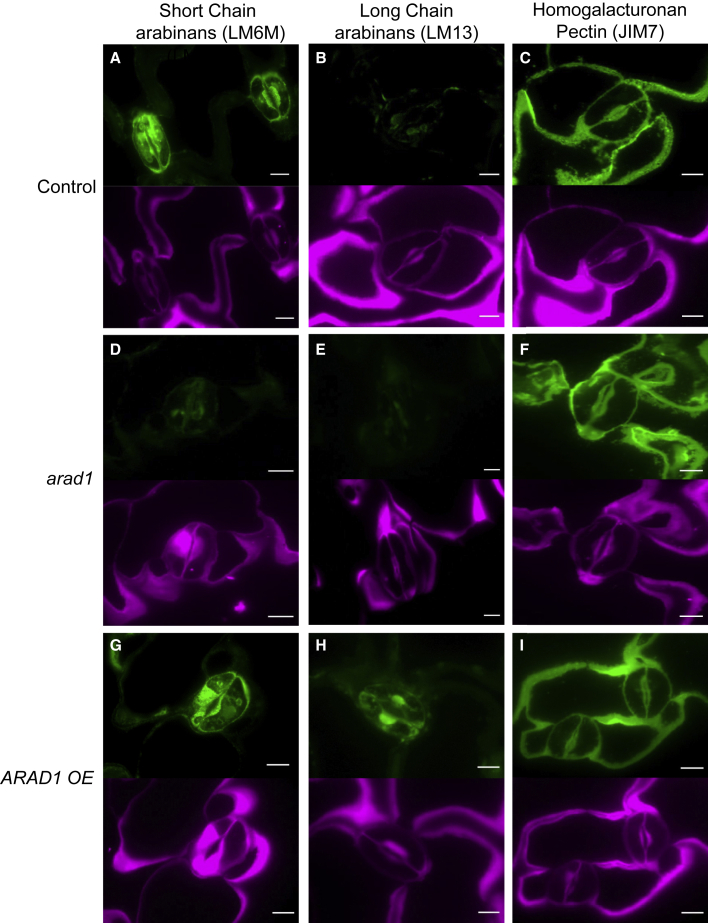
Antibody labeling reveals enrichment of arabinans in guard cells (A–C) Paradermal sections of control tissue incubated with antibodies against SCL-arabinan epitopes (LM6M), LC-arabinan epitopes (LM13), or broad-spectrum pectin (JIM7). (D–F) Paradermal sections of tissue from the *arad1* mutant treated as in (A)–(C). (G–I) Paradermal sections of tissue from ARAD1-OE leaves treated as in (A)–(C). The upper part of each panel shows the antibody signal (green) indicating epitope distribution. The lower part of each panel (purple signal) shows the general distribution of cell wall material revealed by calcofluor staining. Scale bars, 10 μm.

Arabidopsis leaf sections were screened with antibodies (mAb) against a range of cell wall epitopes. This revealed an elevated signal in guard cells for short-chain linear arabinan (SCL-arabinan) epitopes compared to neighboring epidermal cells (LM6M mAb) (Figure 1A, green signal), whereas the signal for longer-chain arabinan epitopes (LC-arabinans; revealed using LM13 mAb) was much lower and did not suggest enrichment in guard cells (Figure 1B). JIM7 mAb binding, which detects a range of homogalacturonan (HGA) polymers within the pectin network, was used as a positive cell wall binding control and was observed across all cell walls in the section (Figure 1C, green signal), supporting the idea that the SCL-arabinan epitope signal reflects a specific guard cell-related pattern of epitope distribution. This was further supported by the pattern of cell wall material shown by calcofluor staining of glucans (Figures 1A–1C, purple signal), which indicated that the patterns observed with LM6M and LM13 did not simply reflect the general distribution of cell wall material in the sections. These data are consistent with previous studies showing guard cell walls of *Commelina communis*, *Vicia faba*, and *Zea mays* are rich in arabinans.^7^^,^^8^ Our data show that Arabidopsis guard cells are specifically enriched in SCL-arabinan epitopes with very few LC-arabinan epitopes present.

The biosynthesis of cell wall arabinans depends upon the function of a set of arabinan synthase-encoding genes.^9^ Previous work has identified two sister genes, *ARABINAN DEFICIENT 1* (*ARAD1*) and *ARABINAN DEFICIENT 2* (*ARAD2*), which are proposed to play a core role in the synthesis of these carbohydrate polymers^9^^,^^10^ (Figure S2A). Analysis of expression databases indicated that while these two genes were not exclusively expressed in the guard cells, transcript levels were enriched in this cell type, notably *ARAD1* (Figures S2B and S2C). We therefore identified and further characterized available mutants for these genes (*arad1* and *arad2*) and generated a double-knockout mutant (*arad1*/*arad2*) (Figures S2D and S2E). When leaf sections of the *arad1* mutant were screened with the same set of antibodies described in Figures 1A–1C, signal with LM6M (SCL-arabinan epitopes) was lost (Figure 1D), again with a lack of signal with LM13 (LC-arabinan epitopes) (Figure 1E), and a relatively uniform cell wall signal was observed with the JIM7 Ab (HGA) (Figure 1F). Similar experiments with *arad2* did not reveal any loss of signal in guard cells incubated with LM6M, and the epitope patterns observed in the *arad1/arad2* mutant were similar to those observed in the *arad1* mutant alone (Figures S1A–S1F).

To confirm these data, we quantified the relative fluorescence signal of the various antibodies used (LM6-M, LM13, and JIM7) in sections for the range of genotypes analyzed (*col-0*, *qrt1*, *arad1*, *arad2*, and *arad1/2*). These data (Figures S1J–S1L) generally corroborated the image data shown in Figures 1 and S1. In particular, there was a loss of LM6-M signal in the *arad1* and *arad1/2* lines with a marginal change in the *arad2* line (Figure S1J). The pattern for LM13 was similar, although there was a relatively high signal in the *arad1*/*arad2* line (Figure S1K), with the JIM7 signal indicating no major difference in signal between any of the lines (Figure S1L). In addition, we performed an ELISA analysis on sequential extractions of wall material to further corroborate the change in arabinan epitopes patterns revealed by our immunolabeling experiments. As shown in Figures S1M–S1O, these data also indicated that there were increases in the level of shorter-chain arabinan epitopes (LM6-M) in the ARAD1-OE lines and a lower level in the *arad1* and *arad1/2* mutant lines. A similar pattern of decreased levels of longer-chain arabinan epitopes (LM13) was also observed in these backgrounds (Figures S1P–S1R). Both sets of data indicated that the *arad1/2* double mutant line contained a similar pattern of depleted arabinan accumulation as the *arad1* mutant that was not apparent in either the *qrt1* or *Col-0* controls. Finally, to investigate whether the altered patterns of arabinan were potentially linked to an altered pattern of hydroxycinnamic esters of pectic side chains (as suggested by previous research^8^), we visualized ester autofluorescence under UV illumination; however, no difference was apparent between control and *arad1* mutant guard cells.

Taken together, our data show that the *arad1* knockout mutants have very low levels of guard cell wall arabinan and suggest that the *ARAD2* gene is not essential for the synthesis of guard cell wall arabinan. This is consistent with previous work showing that *arad2* knockouts display an altered phenotype only in root tissues.^9^

### Loss of SCL-arabinans impairs stomatal opening and decreases conductance

**Figure 2:**
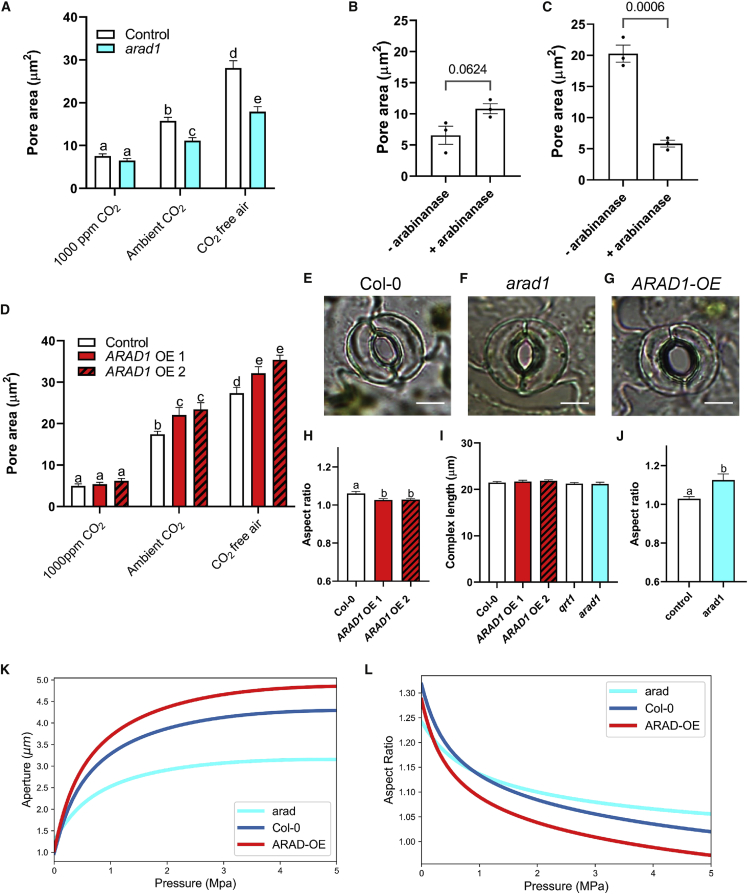
Guard cell arabinans set maximal stomatal aperture (A–C) Bioassay data for (A) control (*qrt1*) (open bars) and *arad1* (cyan) epidermal strips under different CO_2_ levels, as indicated. Columns indicate mean value and error bars = SEM (n = 6). A two-way ANOVA followed by Šidák test was performed. Samples that cannot be distinguished from each other at p < 0.05 are indicated by the same letter. (B) *Col-0* epidermal strips pre-treated with arabinanase and observed under closing conditions (high CO_2_) or (C) under opening conditions (CO_2_ free air). Each point represents the mean aperture observed in a biological replicate (n = 3), with columns indicating mean value and error bars = SEM. Unpaired t tests were performed, and the calculated p value is shown in (B) and (C). (D) Bioassay data for control (*Col-0*) (open bars) and two *ARAD1-OE* (red) epidermal strips under different CO_2_ levels, as indicated. Columns indicate mean value and error bars = SEM (n ≥ 8). A two-way ANOVA followed by Šidák test was performed. Samples that cannot be distinguished from each other at p < 0.05 are indicated by the same letter. (E–G) Images of open stomata for (E) *Col-0*, (F) *arad1*, and (G) *ARAD1-OE*. (H and J) Aspect ratio, as defined by stomatal complex length/complex width of (H) *ARAD1-OE* and (J) *arad1* stomata. (I) Complex length of *ARAD1-OE*, *arad1*, and controls (*Col-0* and *qrt1*). In (H)–(J), columns indicate mean value and error bars = SEM (n ≥ 6). For (H), data were analyzed by ANOVA followed by a Tukey test. For (I), ANOVA indicated that the samples could not be distinguished from each other at the 0.05 confidence limit. For (J), an unpaired t test mutant versus control was performed. For (H) and (J), samples that cannot be distinguished from each other at p < 0.05 are indicated by the same letter. (K and L) Modeled change in pore aperture with pressure (K) or aspect ratio with pressure (L) for *arad1* (cyan), ARAD1-OE (red), and control (*Col-0*) (dark blue) stomata. See also Table S1.

We then investigated the stomatal phenotype of these loss-of-function mutants. Direct measurement of stomatal apertures in epidermal strip bioassays showed that exposure to elevated CO_2_ (1,000 ppm) led to pore closure and CO_2_-free air led to pore opening in control samples (Figure 2A), as previously reported.^3^^,^^11^ In contrast, stomata in the *arad1* mutant had an impaired opening response to low CO_2_ (p < 0.0001), whereas the closing response to elevated CO_2_ could not be distinguished from the control. It should be noted that the available *arad1* mutant is a stock center SAIL mutant, which, as previously highlighted, are unexpectedly often in the *qrt1* background,^12^ as was the case here. In all experiments reported with *arad1*, we used the appropriate *qrt1* background as the control line. To further investigate the role of cell wall arabinans in stomatal opening, we pre-treated leaf samples with exogenous arabinanase for 2 h prior to testing the response of the stomata to altered CO_2_ levels. Consistent with the *arad1* mutant data, the closing response of *arad1* stomata to elevated CO_2_ did not show a major difference compared to the control following arabinanase treatment (p = 0.0624) (Figure 2B), but the opening response was significantly impaired (p = 0.0006) (Figure 2C).

**Figure 3:**
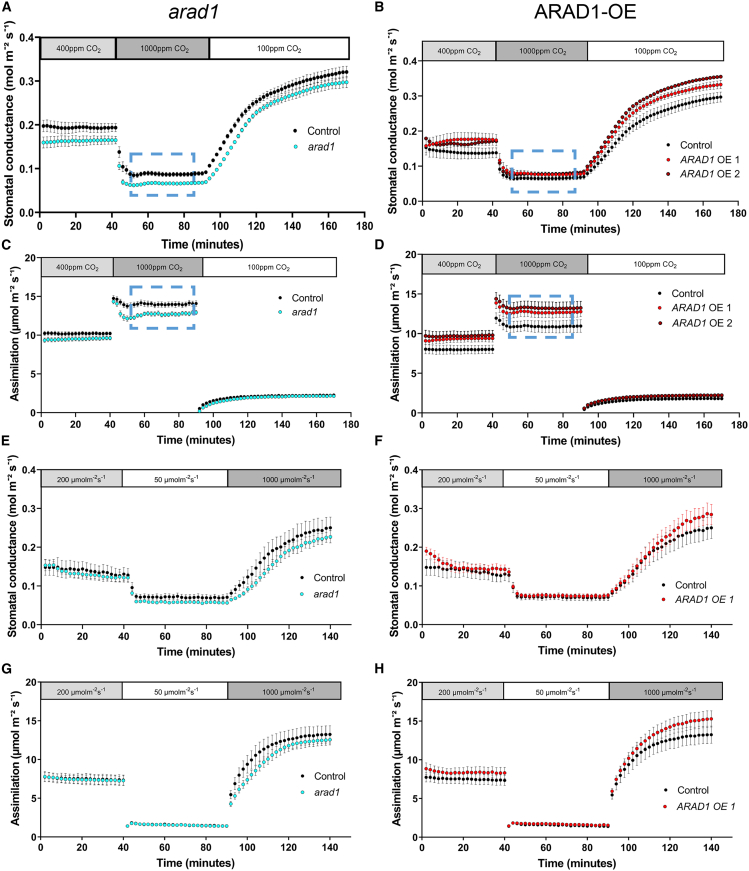
Modulation of maximal stomatal aperture by altered guard cell arabinans leads to altered gas exchange (A–D) Stomatal conductance (A and B) and assimilation rate (C and D) in leaves under a range of CO_2_ concentrations (as indicated) for *arad1* (A and C) and *ARAD1-OE* (B and D) relative to controls. (E–H) Stomatal conductance (E and F) and assimilation rate (G and H) in leaves under a range of irradiance levels (as indicated) for *arad1* (E and G) and *ARAD1-OE* (F and H) relative to controls. n = 5 independent plants, with error bars = SEM.

To test the functional outcome of the loss of *arad1* at the whole-leaf level, infra-red gas exchange analysis was used. Under near-ambient CO_2_ level (400 ppm), *arad1* leaves had a lower stomatal conductance, *g*_*s*_, than control leaves (Figure 3A). This lower *g*_*s*_ was maintained under both elevated and decreased CO_2_ levels. The lower *g*_*s*_ values at a range of CO_2_ levels were reflected in a lower assimilation rate, although at low CO_2_ (100 ppm) the difference was marginal (Figure 3C). In addition to CO_2_, stomatal aperture is known to respond to irradiance level.^11^ Under each of the three irradiances tested (50, 200, and 1,000 μmol m^2^ s^−1^), the *arad1* leaves showed a lower *g*_*s*_ relative to the control leaves (Figure 3E). With respect to assimilation rate, particularly under high irradiance, a condition expected to promote maximal stomatal aperture, there was a decrease in the *arad1* leaves compared to the control (Figure 3G). These differences were not related to changes in stomatal density or index in the *arad1* mutant (Figures S3A and S3C) and did not reflect any difference in theoretical anatomical *gs*_max_ (Figure S3G) or underlying photosynthetic performance, as analyzed by gas exchange analysis (Figure S3E). Thermal imaging of the *arad1*, *arad2*, and *arad1/arad2* double mutant indicated that plants with the *arad1* background had a higher rosette temperature than either the control or *arad2* background (Figures S3H and S3I), consistent with loss of *arad1* having a major outcome on guard cell function.

In our analyses, no stomatal phenotype was observed with the *qrt1* control line, suggesting that the *arad1* allele had the major influence on the phenotype observed, although we cannot fully discount some contribution from the *qrt1* background. Taken together, our analysis of leaves lacking *ARAD1* expression or treated with arabinanase indicated that loss of arabinans in the guard cells led to stomata that had a smaller stomatal aperture and lower gas flux, especially under conditions known to promote maximal pore opening.

### Overexpression of arabinan synthase leads to increased stomatal opening

To investigate the outcome of increasing arabinan synthesis on stomatal function, we created transgenic Arabidopsis plants in which the *ARAD1* gene was ectopically overexpressed (*ARAD1-OE*; Figure S2F). Immunolabeling of *ARAD1-OE* leaves revealed an enrichment of SCL-arabinan epitopes (LM6M) in stomata relative to pavement cells (Figure 1G), as also observed in control samples (Figure 1A). However, LM13 labeling revealed that there was a marked increase in LC-arabinan epitopes in guard cells compared to control samples (Figure 1H). Labeling of HGA and glucans by JIM7 and calcofluor staining (Figures 1G–1I) suggested that the guard cell-enriched signals observed with LM6M and LM13 mAbs were not simply due to altered distribution of total cell wall material. We again corroborated these image data by quantifying the relative fluorescence signal in immunolabeled sections (Figures S1J–S1L). For LM6-M and LM13, there was an increase in signal relative to the Col-0 control in both ARAD1-OE lines analyzed, with no overt change in signal with the JIM7 control antibody. ELISA of cell wall extracts also supported an increased level of arabinan epitopes in the ARAD1-OE lines (Figures S1M–S1R).

The increased accumulation of LC-arabinan epitopes in the guard cells of *ARAD1-OE* leaves was associated with an altered performance in stomatal bioassays. Under conditions promoting stomatal opening (CO_2_-free air), the maximal pore area observed in both *ARAD1-OE* lines analyzed was greater than that observed in the control line (p < 0.0001) (Figure 2D). This difference was maintained under near-ambient levels of CO_2_ (p = 0.004), but under conditions of high CO_2_ (which promote stomatal closure) the *ARAD1-OE* stomata could not be distinguished from the control (Figure 2D). Closer examination of the stomata in *ARAD1-OE* lines under opening conditions revealed an apparent shape change, with the *ARAD1-OE* stomata appearing to be rounder than those in either control or *arad1* leaves (Figures 2E–2G). Measurement of the length-to-width aspect ratio confirmed this, with ARAD1-OE stomata having a lower aspect ratio than control leaves (p = 0.0244) (Figure 2H). In contrast, open *arad1* stomata had a greater aspect ratio than control leaves (p = 0.0154) (Figure 2J). This altered shape was not reflected in any difference in the length of the stomatal complexes (Figure 2I), suggesting that there was an altered lateral flexing of the guard cells in the mutant stomata.

### Computational modeling and AFM analysis indicate changes in stiffness after modulation of guard cell wall arabinans

In previous work, we reported on the generation of a finite element (FE) model of guard cell mechanics that successfully recapitulated aspects of stomatal movement in response to turgor change.^5^^,^^13^ To investigate how a change in cell wall arabinan composition might mechanically influence guard cell performance to capture the phenotypes reported here, we explored parameter space to identify changes in the model that might plausibly simulate the observed changes in stomatal opening/closure in the *arad1* and *ARAD1-OE* lines. Starting with the base model,^13^ we matched the model parameters to the mean dimensions of stomatal pore size and shape, and stomatal complex dimension measured in *arad1*, *ARAD1-OE*, and control plants (Table S1), then empirically explored whether parameters linked to either the modeled cellulose fiber network or the wall matrix led to outputs similar to the shape changes observed in these mutants. Altering fiber stiffness (with no other alteration to anisotropy or wall matrix properties) led to only minor changes in the pressure/aperture response curves. However, decreasing the stiffness of the cell wall matrix was sufficient to stretch the pressure/aperture response curve so that a greater final aperture was achieved (red line in Figure 2K; Table S1), similar to the phenotype observed in the *ARAD1-OE* transgenic plants. Conversely, increasing the Young’s Modulus, E, of the cell wall matrix led to a dampening of the aperture/pressure response curve, with a smaller final aperture at high pressure, recapitulating the phenotype of the *arad1* mutant stomata (blue line in Figure 2K; Table S1). Reflecting the observed measurements used to parameterize the model, the stomatal apertures at low turgor pressure were similar in all cases. With respect to aspect ratio, at higher turgor pressure the model predicted a smaller aspect ratio (more circular stomata) in the *ARAD-OE* line and a higher aspect ratio (more elliptical stomata) in the *arad1* mutant, successfully capturing this aspect of the phenotype (Figure 2L).

**Figure 4:**
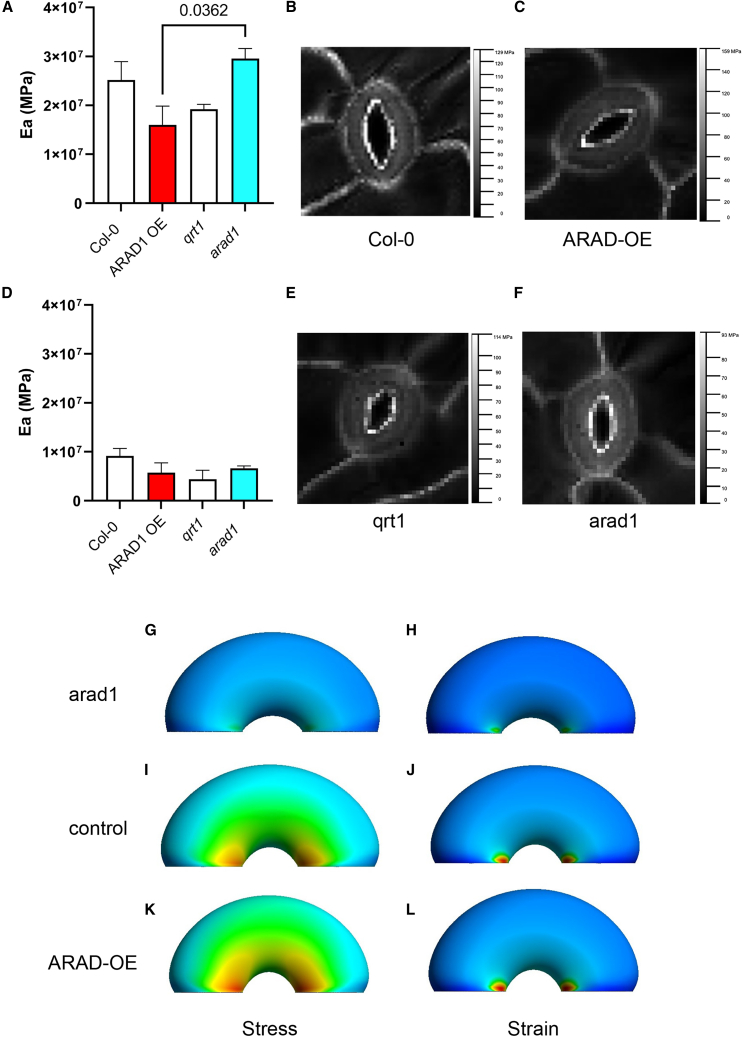
Altered arabinans in the guard cell leads to altered wall stiffness (A) Measured apparent Young’s modulus, E_a_, in guard cells of *col-0*, *ARAD-OE* (red), *qrt1*, and *arad1* (cyan) leaves. Columns indicate mean values for a pair of guard cells, with error bars = SEM and n ≥ 3. ANOVA followed by a Tukey test indicated that the mean E_a_ value for *arad1* could be distinguished from that for *ARAD1-OE* (p = 0.0362). (B and C) Force maps of stomata from (B) *Col-0* and (C) *ARAD-OE*. (D) Measured E_a_ in epidermal cells adjacent to guard cells of *Col-0*, *ARAD-OE* (red), *qrt1*, and *arad1* (cyan) leaves. Columns indicate mean values. Error bars = SEM with n = 3. ANOVA indicated that none of the samples could be distinguished from each other at the 0.05 confidence limit. (E and F) Force maps of stomata from (E) *qrt1* and (F) *arad1* plants. (G–L) Modeled outputs of stress (G, I, and K) and strain (H, J, and L) for *arad1* (G and H), control (I and J), and *ARAD-OE* (K and L) guard cells undergoing opening. The stress plots display the effective Cauchy stress (MPa = 10^6^ N/m^2^) and the strain plots show the effective Lagrange strain (unitless), with warmer colors indicating higher values.

To test whether the mechanical properties of guard cells were altered in the *arad1* and *ARAD-OE* lines, we performed a series of atomic force microscopy (AFM) measurements, using a similar approach to that previously described.^5^ The results indicated that the apparent modulus (E_A_) of the *arad1* guard cells was significantly higher than that calculated for the *ARAD1-OE* guard cells (Figure 4A) (p = 0.0362), consistent with the shift required by the computational modeling summarized in Table S1. The relative increase in E_A_ between *arad1* and *ARAD1-OE* measured by AFM (2.4×) was of a similar magnitude to that predicted by the model (1.8×), again suggesting that the model was capturing a realistic element of guard cell mechanics. Analysis of E_A_ values in the pavement cells of the same samples did not reveal any statistically significant difference between the different genotypes (Figure 4D), suggesting that the changes observed in the guard cells were having the major effect on the mechanical behavior of the system. Analysis of the AFM force maps did not reveal any overt differences in the spatial distribution of E_A_ between the lines analyzed, consistent with a view of altered arabinan composition leading to bulk changes in guard cell wall stiffness to underpin the observed phenotype (Figures 4B, 4C, 4E, and 4F).

The FE modeling approach allows estimation of stress/strain patterns within the system at various points along the curves shown in Figure 2K. Considering stomata at high turgor pressure, the *arad1* guard cells (Figures 4G and 4H) are predicted to experience relatively low stress and strain compared with the modeled *Col-0* cells (Figures 4I and 4J), with *ARAD1-OE* cells showing slightly higher stress/strain values than *Col-0* (Figures 4K and 4L). In all cells there is a radial gradient of stress/strain emanating from the inner point of the guard cell tip edge, but the model outputs suggest that the accumulation of long-chain arabinans leads to the inner walls of the *ARAD-OE* guard cells accommodating higher stress levels than the *arad1* mutant while undergoing greater radial bending (compare Figures 4G and 4K).

### Increased LC-arabinans in the guard cell wall leads to increased stomatal gas exchange

To investigate the outcome of the altered stomatal properties observed in the ARAD1-OE plants at the whole-organ level, we performed a series of gas exchange analyses (Figure 3B, 3D, 3F, and 3H). In contrast to *arad1*, leaves of *ARAD1-OE* showed a higher *g*_*s*_ than control leaves under low and near-ambient CO_2_ conditions, although under elevated CO_2_ levels (expected to close stomata) this difference was not so marked (Figure 3B). The increased level of *g*_*s*_ was reflected in a higher assimilation rate at all CO_2_ levels, though the difference under low CO_2_ was marginal (Figure 3D). Under differing irradiance levels, stomatal conductance in the *ARAD1-OE* leaves was slightly higher than that observed in control leaves, and at higher light levels (expected to maximally open stomata) this difference was more marked (Figure 3F). These differences in *g*_*s*_ under different light regimes were reflected in the assimilation rates, with markedly higher rates being observed under high light conditions (Figure 3H). Our analysis indicated that the measured differences in *g*_*s*_ did not reflect any significant difference in theoretical *g*_*s*max_ (Figure S3G) or underlying photosynthetic performance of the leaves, as analyzed by gas exchange analysis (Figure S3F).

One interesting observation from the gas exchange analyses shown in Figure 3 was that under conditions of elevated CO_2_, the assimilation rate of the *ARAD1-OE* line was higher than the control, whereas stomatal conductance under these conditions was similar for both transgenic and control (boxed regions in Figures 3B and 3D). To investigate this further, we calculated the intrinsic and instantaneous water use efficiency (iWUE and WUE) for leaves of *arad1* and *ARAD1-OE* lines and compared them with the relevant controls under the range of CO_2_ conditions shown in Figure 3. As shown in Figure S3J, iWUE and WUE were slightly higher in *arad1* leaves relative to controls at all CO_2_ levels (1.1×, 1.1×, and 1.2× control), whereas in *ARAD1-OE* leaves both were lower relative to control values at low and ambient CO_2_ levels (0.85× and 0.9× control, respectively). However, at elevated CO_2_ the iWUE and WUE values for *ARAD1-OE* leaves were higher relative to the control, with the increase being comparable to that calculated for *arad1* leaves (1.1× versus 1.2× control). Under elevated CO_2_ the carbon assimilation rate was lower in the *arad1* leaves compared to control (0.85× control), whereas for *ARAD1-OE* leaves under these conditions there was a relative increase in assimilation rate (1.2× control). Thus, under conditions of elevated CO_2_ the *ARAD1-OE* plants undergo a shift to improved water-use efficiency comparable to *arad1*, but this occurs against a background of a relative increase rather than decrease in carbon assimilation rate.

This study extends previous work suggesting that pectic arabinans are involved in setting guard cell wall properties^7^ and that, more generally, high levels of arabinan are associated with increased tissue flexibility.^14^^,^^15^ We provide functional genetic and biochemical evidence that demonstrate that arabinan composition dictates the parameters of stomatal opening/closing by modulating wall, and thus cell, flexibility. We show that shorter linear arabinan chains lead to a stiffer, less flexible guard cell wall, while longer chains lead to a less stiff wall with increased flexibility. Computational modeling, backed up by AFM measurements, suggests a plausible underpinning mechanism whereby a uniform influence on the guard cell wall Young’s modulus via altered arabinan composition leads to the specific cellular behavior observed. Exactly how arabinan chain length influences wall mechanical properties awaits elucidation. In addition, although our data strongly implicate *ARAD1* as the key player in guard cells, we cannot exclude a role of *ARAD2* in this process. Classical models of cell wall structure/function have come under scrutiny, with a body of data supporting models in which the degree of polymer entanglement plays an important role in setting matrix mechanical properties.^16^, ^17^, ^18^ In such models, decreasing the amount of short linear arabinan chains (as in the *arad1* mutant) could promote a higher degree of polymer entanglement (leading to a stiffer matrix), while introducing longer arabinan chains (as in the *ARAD1-OE* lines) might act to physically distance load-bearing elements of the matrix, decreasing the degree of entanglement, thus decreasing wall stiffness. Other pectin modifications (e.g., demethylation patterns) might be super-imposed on the pattern/degree of polymer entanglement, influencing the ability of stretches of homogalacturan polymers to form, e.g., calcium bridges, thus further modulating wall mechanics and accounting for several reported outcomes of pectin modification on guard cell function.^3^^,^^4^^,^^6^ The development of more advanced methods of probing wall structure,^19^ coupled with continued advances in the application of methods to measure plant cell wall mechanical properties,^20^, ^21^, ^22^ provide the means to test such ideas.

Overall, the data presented here add to our understanding of how guard cell walls achieve the unique combination of strength and flexibility required as they undergo repeated extreme changes in shape while withstanding major changes in turgor pressure.^23^ In addition to the geometric properties provided by cellulose anisotropy and polar stiffening^2^ and the central role of pectins,^3^^,^^4^^,^^6^ modulation of wall modulus by arabinan side chains helps set the upper dynamic limit of the system. Our work further highlights the utility of the guard cell as a test bed for the elucidation of wall structure and function.

Finally, the mechanical properties of guard cells contribute to the dynamics and degree of stomatal opening^5^^,^^24^ and, therefore, play an important role in whole-plant water relations, crop stress resilience, and yield protection.^25^ Following success via manipulating stomatal density,^26^^,^^27^ improving crop stomatal function via targeted modification of guard cell walls is an attractive biotechnology target. Our data show that increasing guard cell LC-arabinan epitopes allows stomata to open to a greater extent but does not compromise the closing response. Gas exchange analysis indicates that although under present ambient CO_2_ levels plants with increased levels of LC-arabinan epitopes in their guard cells would have a lower WUE due to the altered dynamics of stomatal opening/closing, under elevated CO_2_ the change in WUE would be minimal and yet would come with the benefit of increased carbon assimilation, opening the door to potential increased yield for comparable water use. We suggest that the manipulation of guard cell flexibility via *ARAD1* in crops could provide higher yields in a future scenario of further increases in atmospheric CO_2_ level.

## STAR★Methods

### Key resources table

**Table:** 

REAGENT or RESOURCE	SOURCE	IDENTIFIER
**Antibodies**		

JIM7	http://www.plantprobes.net/index.php	JIM7
LM6M	http://www.plantprobes.net/index.php	LM6M
LM13	http://www.plantprobes.net/index.php	LM13
Anti-rat-IgG-FITC	Thermofisher	31629; RRID: AB_228240
Anti-rat-IgG-HRP	Thermofisher	A10549; RRID: AB_2534047

**Bacterial and virus strains**		

Agrobacterium GV3101 pMP90	GoldBio	CC-207

**Chemicals, peptides, and recombinant proteins**		

Calcofluor white	Merck	18909
Endo-arabinase	Megazyme	E-EARAB
LR White Resin	London Resin Company	AGR1280

**Experimental models: Organisms/strains**		

Arabidopsis thaliana: col-0, qrt1, arad1	NASC; https://www.arabidopsis.org/portals/mutants/stockcenters.jsp	Col-0, qrt1, arad1, arad2
ARAD1-OE1 and ARAD1-OE2	This Manuscript	*ARAD1-OE*
Arad1/arad2	This Manuscript	*Arad1/2*
Agrobacterium tumefaciens: GV3101 pMP90	https://www.goldbio.com	CC-207

**Oligonucleotides**		

*arad1* genotyping Fwd primer TATGTGTTCAGGGTGGAAAAGT	This Manuscript	N/A
*arad1* genotyping Rev primer GGGAGACTTGACGCCAGATT	This Manuscript	N/A
*arad2* genotyping Fwd primer TCGTTTATTTTGGTGGCAGTC	This Manuscript	N/A
*arad2* genotyping Rev primer CGCCTCAGCCGGGTCAAAA	This Manuscript	N/A
Sail LB primer GCCTTTTCAGAAATGGATAAATAGCCTTGCTTCC	This Manuscript	N/A
Fwd primer for amplification of ARAD1 coding sequence caccATGGCGCGTAAATCTTCCCTCCTCAAAC	This Manuscript	N/A
Rev primer for amplification of ARAD1 coding sequence TTAAATGGAAGTGATAAGACCGGTTTGG	This Manuscript	N/A
RT PCR primer for *arad1* FWD: GCTCCTCCACAGTCCAAAAG	This Manuscript	N/A
RT PCR primer for *arad1* RED: ACGAGCTGCTACGAAAGGAA	This Manuscript	N/A
RT PCR primer for *ARAD1-OE* FWD: GAGTTGAGGATCGCAACACA	This Manuscript	N/A
RT PCR primer for *ARAD1-OE* REV: CGTAGCAGCTCGTCGATTCT	This Manuscript	N/A

**Recombinant DNA**		

Plasmid: pENTR-D-TOPO	Thermofisher	K240020
Plasmid:PMDC32	Addgene	32078

**Software and algorithms**		

FEBIO	FEBio Software Suite	https://febio.org
Finite Element Stomatal Model	GitHub	https://github.com/woolfeh/stomasimulator; http://doi.org/10.5281/zenodo.6546037
ImageJ (Fiji)	NIH (Public Domain)	https://imagej.nih.gov/ij/

### Resource availability

#### Lead contact

Further information and requests for resources and reagents should be directed to and will be fulfilled by the lead contact, Andrew Fleming (a.fleming@sheffield.ac.uk).

#### Materials availability

All newly created *Arabidopsis* lines and the vectors used in their creation can be obtained by contacting the lead contact.

### Experimental model and subject details

Arabidopsis seeds (*Col-0*, *qrt1*, *arad1*, *arad2*, *arad1/arad2, ARAD1-OE1, ARAD1-OE2*) were sown directly onto 60x60x80mm pots of pre-soaked M3 compost and perlite (3:1). The pots were stratified at 4°C for 1 week before being transferred to a controlled environment growth chamber (12 h light/12 h dark, 200 μmol m^-2^ s^-1^ PPFD, 22°C light/ 16°C dark, 60% humidity). The seedlings were thinned to 2 plants per pot at 10 days and further to 1 plant per pot at 14 days. Plants were harvested for experimental work after 5 weeks growth.

### Method details

#### Molecular biology

The *arad1* (SAIL_189_F10) and *arad2* (SAIL_881_C10) T-DNA insertion lines were obtained from NASC (Nottingham, UK) and confirmed as homozygous for the insertion by PCR using the primers: arad1 5’-TATGTGTTCAGGGTGGAAAAGT- and 5’-GGGAGACTTGACGCCAGATT- arad2 5’-TCGTTTATTTTGGTGGCAGTC- and 5’-CGCCTCAGCCGGGTCAAAA- and the SAIL LB primer 5’-GCCTTTTCAGAAATGGATAAATAGCCTTGCTTCC-. The *arad1/arad2* line was created by crossing the *arad1* and *arad2* T-DNA insertion lines, with homozygous T3 seed used for phenotypic characterization.

For the *ARAD1-OE* line the ARAD1 coding sequence was amplified from gDNA using primers 5’- caccATGGCGCGTAAATCTTCCCTCCTCAAAC – and 5’- TTAAATGGAAGTGATAAGACCGGTTTGG- and recombined into the pENTR-D-TOPO vector (Invitrogen). This was then cut by NsiI (NEB) and recombined by LR reaction into pMDC32. Plasmids were transformed into the GV3101 pMP90 agrobacterium strain by electroporation and transformed into the Col-0 background by floral dip.^28^ Transformants were selected on 0.5X MS (Murashige and Skoog) medium, 1.5% (w/v) sucrose containing 50mg/mL kanamycin and plants from the T3 generation were analyzed.

Gene expression levels were verified by RT-PCR following RNA extraction using the Spectrum RNA extraction kit as per manufacturer instructions. The following primer pairs were used: *arad1* 5’- GCTCCTCCACAGTCCAAAAG- and 5’- ACGAGCTGCTACGAAAGGAA-, ARAD1-OE 5’- GAGTTGAGGATCGCAACACA- and 5’- CGTAGCAGCTCGTCGATTCT.

#### Stomatal aperture measurements

Epidermal peels of mature leaves were removed at least 2 hours into the photoperiod and floated onto opening buffer (50 mM KCl, 10 mM MES, pH 6.2). Samples were maintained at 22°C with 200 mmol m^-2^ s^-1^ of light. Air was bubbled into the opening buffer containing either 0 ppm CO_2_ (CO_2_ free treatment), ambient CO_2_ or elevated CO_2_ (1000 ppm). Epidermal peels were imaged after 2 hours using an Olympus BX51 microscope and DP70 digital camera and stomatal apertures measured. For standard assays, 42 stomatal apertures were measured for each treatment in each of three independent experiments, with similar results being observed in each experiment. For each experiment epidermal peels were taken from at least 6 plants of each genotype. For the arabinanase pre-treatment epidermal peels were taken from plants and floated onto buffer (10 mM MES (pH6.2), 10 mM KCl, 0.1 mM CaCl2) containing 10 units/mL arabinanase (endo-/exo-Arabinanase from *Cellvibrio japonicus*, Megazyme) for 1 h before being introduced into the bioassay system.

#### Infrared gas exchange analysis

CO_2_ shifts and light shifts were carried out on 5-week-old plants. Measurements were taken with a LICOR-6800 infrared gas exchange analyzer with a 6 cm^2^ circular area for measurement. If the leaf did not fill the chamber the leaf area was measured and corrected for in the analysis. For both experimental set ups leaf temperature was maintained at 21°C, humidity at 60%, flow rate at 300 μmol s^-1^ and fan speed 10,000 rpm. For CO_2_ shifts photon flux density was maintained at 300 μmol m^-2^ s^-1^ with 10% blue light. For light shifts CO_2_ was maintained at 400 ppm.

For CO_2_ shifts a mature leaf was clamped into the chamber and allowed to acclimate at 400 ppm CO_2_ until stomatal conductance and assimilation rates had stabilized. The CO_2_ shift then began by supplying the leaf with 400 ppm CO_2_ for 40 min, followed by 1000 ppm CO_2_ for 50 min and finally 100 ppm CO_2_ for 80 min. Gas exchange measurements were recorded every 2 min throughout the experiment. Intrinsic water-use efficiency (iWUE) was calculated by dividing the assimilation rate by the stomatal conductance at a given time point and WUE (WUE) was calculated by dividing the assimilation rate by the transpiration rate at a given time point.

For light shifts a mature leaf was clamped into the chamber and allowed to acclimate at a photon flux density of 200 μmol m^-2^ s^-1^ with 10% blue light until the stomatal conductance and assimilation rate had stabilized. The light shift was then started with 200 μmol m^-2^ s^-1^ light for 40 min, followed by 50 μmol m^-2^ s^-1^ light for 50 min and finally 1000 μmol m^-2^ s^-1^ light for a further 50 min (all 10% blue light). Gas exchange measurements were taken every 2 min throughout the experiment.

#### Analysis of stomatal number

For stomatal density analysis fully expanded non-senescent leaves were harvested from 5 week old plants. Leaves were fixed in 4% (v/v) formaldehyde in PEM buffer (0.1 M PIPES, 2 mM EGTA, 1 mM MgSO4, adjusted to pH 7) for 8 hours. Leaves were then washed twice in 70% (v/v) ethanol for 30 minutes each wash. Tissue was then cleared by incubation in chloral hydrate (2.5 g mL-1) in 30% (v/v) glycerol twice for 8 h. Samples were then mounted in 30% (v/v) glycerol solution and imaged on an Olympus BX51 microscope under the 40x objective using Nomarski illumination, images were captured with an Olympus DP70 camera and the number of stomata counted. 4 viewpoints per leaf were analyzed.

#### Immunolabeling

For immunolabeling, leaf samples (3mm diameter leaf discs) were fixed in 4% (w/v) formaldehyde in PEM buffer (0.1MPIPES, 2mM EGTA, 1 mM MgSO4, adjusted to pH 7) by vacuum infiltration then dehydrated in an ethanol series (30 min each at 30%, 50%, 70%, 100% EtOH) and infiltrated with LR White Resin (London Resin Company) diluted in ethanol (45 min each at 10%, 20%, 30%, 50%, 70% & 90% resin then 3x8 h at 100%). Leaf discs were stacked vertically in gelatine capsules filled with resin and allowed to polymerize for 7 days at 37°C. Sections were cut to a thickness of 2 mm using a Reichert-Jung Ultracut E ultramicrotome using a glass knife. Further processing and incubation with the JIM7 and LM19 antibodies was as previously described.^3^ Briefly, sections were incubated with 3% (w/v) milk protein (Marvel, Premier Beverages, UK) in phosphate-buffered saline solution (PBS, pH 7.2) (hereafter known as PBS/MP). Sections were then incubated with a ten-fold dilution of primary monoclonal antibody in PBS/MP for 1 h at room temperature. Samples were washed 3 times with PBS and secondary antibody was added (anti-rat-IgG (whole molecule) coupled to fluorescein isothiocyanate FITC was used at 100-fold dilution in PBS/MP) for 1 h. Samples were kept in the dark from this step. Samples were counterstained with 0.25% (w/v) Calcofluor White solution diluted ten-fold in PBS for 5 min before mounting on slides with Citifluor AF1 anti-fade solution (Agar Scientific, UK). Images were captured using a DP51 camera. FITC was visualized using a filter set with 460-490 nm excitation filter, a 510-550 nm emission filter and a 505 nm dichroic mirror. Calcofluor White was visualized using a 395 nm excitation filter, a 460 nm emission filter and a 425 nm dichroic mirror.

#### Preparation of alcohol insoluble residues

Leaf tissue was flash frozen and freeze dried before being ground to a fine powder in a Qiagen TissueLyserII (Qiagen, Hilden, Germany) at 30Hz for 1 minute with two 3mm stainless steel ball bearings. To extract alcohol insoluble resides (AIR) 250 mg of ground tissue was sequentially incubated in 1 ml volumes of a solvent series consisting of ethanol (50%, 60%, 70%, 80%, 90% and 100% v/v) followed by acetone and a chloroform:methanol mixture (3:1) at each stage samples were incubated for 90 minutes on a rocking table at room temperature, sample pelleted by centrifugation and the solvent discarded. Following the final step, the sample was dried by evaporation leaving AIR which is enriched in cell walls.

#### ELISA of cell wall extracts

Cell walls were sequentially fractionated by the following procedure. 2 mg of AIR was incubated 1 ml of with 50 mM Cyclohexanediaminetetraacetic acid (CDTA), pH6 for 90 minutes and shook at 10 Hz in a Qiagen TissueLyserII. Undissolved sample was pelleted by centrifugation and the supernatant retained as the CDTA extract. This extraction process was repeated with 4 M Potassium Hydroxide and retained as the KOH extract. Any remaining residues were subjected to a cellulase digestion by incubating for 8 hours at 30°C with 1μg/ml of cellulase 5A (NZYTech, Lisbon, Portugal) in 20 mM Tric-HCl buffer pH 8.8 to give the cellulase extract. 20μl of each extract was diluted 5x using using 0.01 M Phosphate Buffer Saline (PBS), pH 7.4 in an immunosorp 96-well plate (Maxisorp, F96, Thermofisher) and incubated overnight at 4°C to coat the plates. After coating the plates were rinsed in tap water and blocked for 1 h at room temperature using 200 μL PBS containing 5% (w/v) nonfat bovine milk powder (Sigma) followed by extensive washing with tap water. 100 μL primary antibody (10x diluted in PBS containing 5% (w/v) milk powder) was added and incubated for 2 h at room temperature. Again the plates were washed, and 100 μL of anti-rat IgG coupled to horseradish peroxidase (Thermofisher), 1000-fold dilution in PBS containing 5% (w/v) milk powder, was added and incubated for 1 h followed by another washing step.

The plates were developed by adding 100 μL of substrate solution (to make 20 ml of substrate 2 ml of 1M sodium acetate pH 6; 200μl of 10 mg/ml of 3,3′,5,5′-tetramethylbenzidine in DMSO and 20 μl of 6% hydrogen peroxide were added to 17.78 ml of water) and incubate for 6 minutes. The reaction was stopped by adding 50 μL of 2.5 M H_2_SO_4_, resulting in the formation of a yellow color measured at 450 nm.

#### Atomic force microscopy

The method used was based on that described previously.^5^ Dissected and plasmolyzed (0.55 M mannitol; minimum 45 min) leaf blocks (approximately 5mm square) from 3-4 week old plants were indented using a Nano Wizard 3 AFM (JPK Instruments, DE) mounted with a 5 nm diameter pyramidal indenter (Windsor Scientific, UK) on a cantilever of nominal 45 N/m stiffness. Cantilever stiffness was determined by thermal tuning prior to experiment initiation. Tip sensitivity was calibrated by first performing indentations on a clean glass slide and varied between experiments. For each leaf, areas of 100x100 μm^2^ were indented with 128x128 points on the adaxial surface. Indentations were performed with 250 nN of force yielding an indentation depth range of 10-100 nm. Sample numbers for each experiment are given in the figure legends and text. Force indentation curves were analyzed using JPKSPM Data Processing software (JPK Instruments, DE; v. spm 5.0.69) using the following steps: voltage readings were converted to force using calibrated sensitivity and cantilever stiffness values, baseline subtraction and tilt correction, vertical displacement offset adjustment, indentation calculation by subtraction of cantilever bending from piezo position during indentation, and indentation modulus was calculated by fitting a Hertzian indentation model to the approach curve. The Hertz model assumes the indented surface is an infinite homogeneous half space, which is clearly not the case for the geometrically complex leaf surface. Hence the results of indentation experiments are quoted as an apparent modulus, E_a_. Control experiments carried out at lower indentation rates and at lower indentation depths revealed similar results, and analysis did not reveal any surface topography which might easily account for the E_a_ patterns observed around or within the guard cells. Retraction curves were not analyzed due to numerous adhesion difficulties during tip removal from the surface. All AFM images shown are derived from force maps, with an indication of the calculated E_a_ values according to the heatmaps adjacent to the images.

#### Computational modelling

We used the finite element (FE) model of guard cell mechanics that was previously described^5^^,^^13^ and solved with FEBio.^29^ In brief, a guard cell pair is represented as two hollow, deformed tori that form ellipses for the pore and complex outlines. The two guard cells are connected by solid walls at the two poles. The initial geometry of the guard cell complex is described by the stoma length, pore length, pore width and guard cell width. These dimensions are matched to the mean data for *arad1*, *col-0* control and *ARAD1-OE* cell types for the high CO_2_ conditions (Table S1). In all three cell types, the cell wall thickness and polar wall thickness was set to 0.1 and 0.3 μm, respectively, and the polar walls were fixed in space consistent with the findings previously reported.^5^ The guard cell wall was modelled using the transversely isotropic Veronda-Westmann material which is an uncoupled hyperelastic material that exhibits strain-stiffening.^30^ The Veronda-Westmann model for the isotropic cell wall matrix is described by two empirically determined coefficients C1 and C2. C1 (MPa) scales the magnitude of the stress-strain curve and C2 (dimensionless) defines the magnitude and nonlinearity of the stress-strain curve. These two parameters can be related to the Young’s Modulus, a measure of the stiffness of a material, by E=3(C1^∗^C2) which is valid only for small strains. For larger strains, the exponential term determined by C2 dominates and the strain-stiffening behavior is more pronounced with materials of a larger C2 becoming exponentially stiffer with increasing strain. The Bulk Modulus, a measure of a material’s resistance to compressibility, was set to 10 GPa, to make the material nearly incompressible. The Poisson ratio is a measure of the material’s deformation in a direction perpendicular to the load and is ≈0.5 for incompressible materials. The anisotropic direction, representing the circumferential cellulose microfibril (CMF) orientation, was calculated for the ∼18000 elements using an in-house meshing script. As previously reported,^12^ the fiber parameters were reduced to one value, C5 (MPa). In this study, C5 was found to have minimal effect on stomatal geometry and held constant for all cell types. The pressure/aperture graphs were achieved by increasing the pressure load (i.e., turgor pressure) from 0 to 5 MPa using the variable iterator which adjusts the incrementation value depending on the convergence data at the previous step. The stress/strain plots display the effective stress (MPa = 10^6^ N/m^2^) and effective Lagrange strain (unitless) respectively. The effective Lagrange strain (unitless scalar), is calculated from the Lagrange strain tensor, E, and deviatoric strain, E’, where E’=E-(tr(E)/3)^∗^I, where tr(.) is the trace and I is the identity tensor. The effective Lagrange strain, *e*, is then given by $e=\sqrt{\frac{3}{2}\left(E^{\prime }:E^{\prime }\right)}$ where : indicates a double contraction of the tensor. A similar calculation is done for the stress tensor yielding the effective stress (von Mises stress).

### Quantification and statistical analysis

Most of the experiments were comprised of 6 or more biological replicates with number of events measured indicated in the figure legends. Statistical differences were assessed by Student’s t test or ANOVA, using built-in functions of the statistical package GraphPad, with the exact test performed indicated in the figure legends. Statistical significance is indicated on the graphs and in the figure legends. Error bars represent standard error.

## Data Availability

All of the scripts used to run these simulations, process the data and generate the graphs are available at Github: http://doi.org/10.5281/zenodo.6546037.

## Supplemental information

Document S1. Figures S1–S3 and Table S1

Document S2. Article plus supplemental information
