# Altering arabinans increases *Arabidopsis* guard cell flexibility and stomatal opening

**DOI:** 10.1016/j.cub.2024.05.035

**Published:** 2024-06-17

**Authors:** Sarah Carroll, Sam Amsbury, Clinton H. Durney, Richard S. Smith, Richard J. Morris, Julie E. Gray, Andrew J. Fleming

## Main text

(Current Biology *32*, 3170–3179.e1–e4; July 25, 2022)

After the original publication, we noted that an error had occurred during article preparation, leading to the incorporation of an earlier version of Figure 4 than was reviewed and accepted for publication. This earlier version of the figure contains formatting errors and does not completely concur with the text describing it. This mistake has been corrected online; the correct version of Figure 4 is now included in the paper. The figure legend and accompanying text describing and interpreting the results remain the same and are correct. The authors apologize for this mistake and the confusion it may have caused.

**Figure 4:**
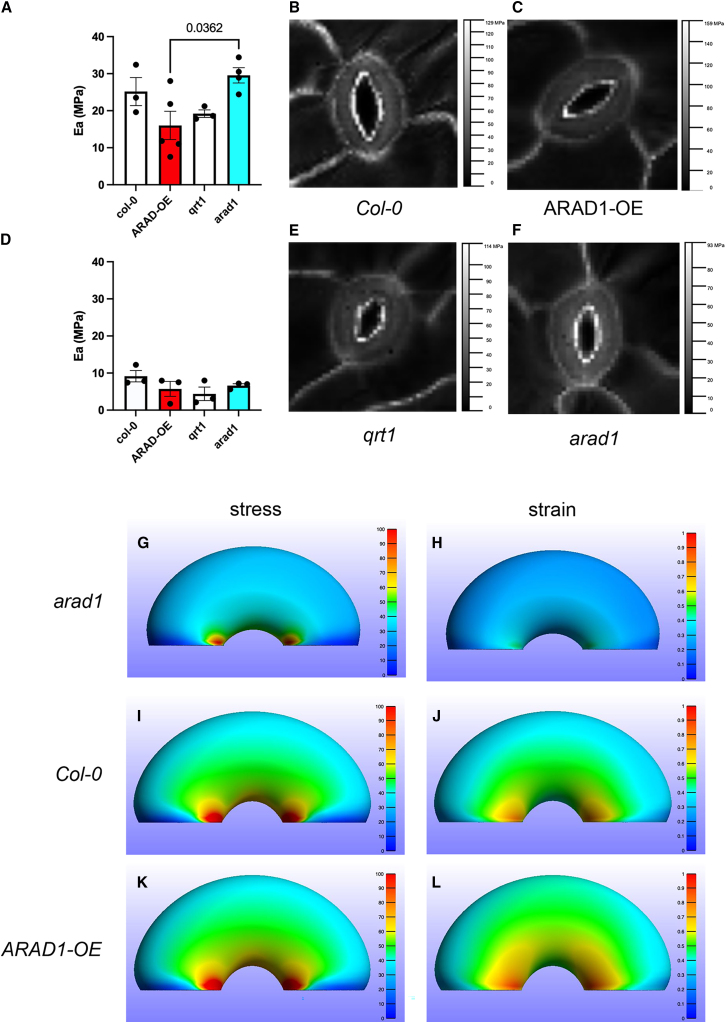
Altered arabinans in the guard cell leads to altered wall stiffness (corrected)

