# Supplementary material for: Altering arabinans increases Arabidopsis guard cell flexibility and stomatal opening
Source: Curr Biol. 2022 Jul 25;32(14):3170–3179.e4. doi: 10.1016/j.cub.2022.05.042 (PMC9616722; doi:10.1016/j.cub.2022.05.042)
Supplement: Document S1. Figures S1–S3 and Table S1 [file mmc1.pdf]

Current Biology, Volume *32*

Supplemental Information

Altering arabinans increases *Arabidopsis* guard cell flexibility and stomatal opening

Sarah Carroll, Sam Amsbury, Clinton H. Durney, Richard S. Smith, Richard J. Morris, Julie E. Gray, and Andrew J. Fleming

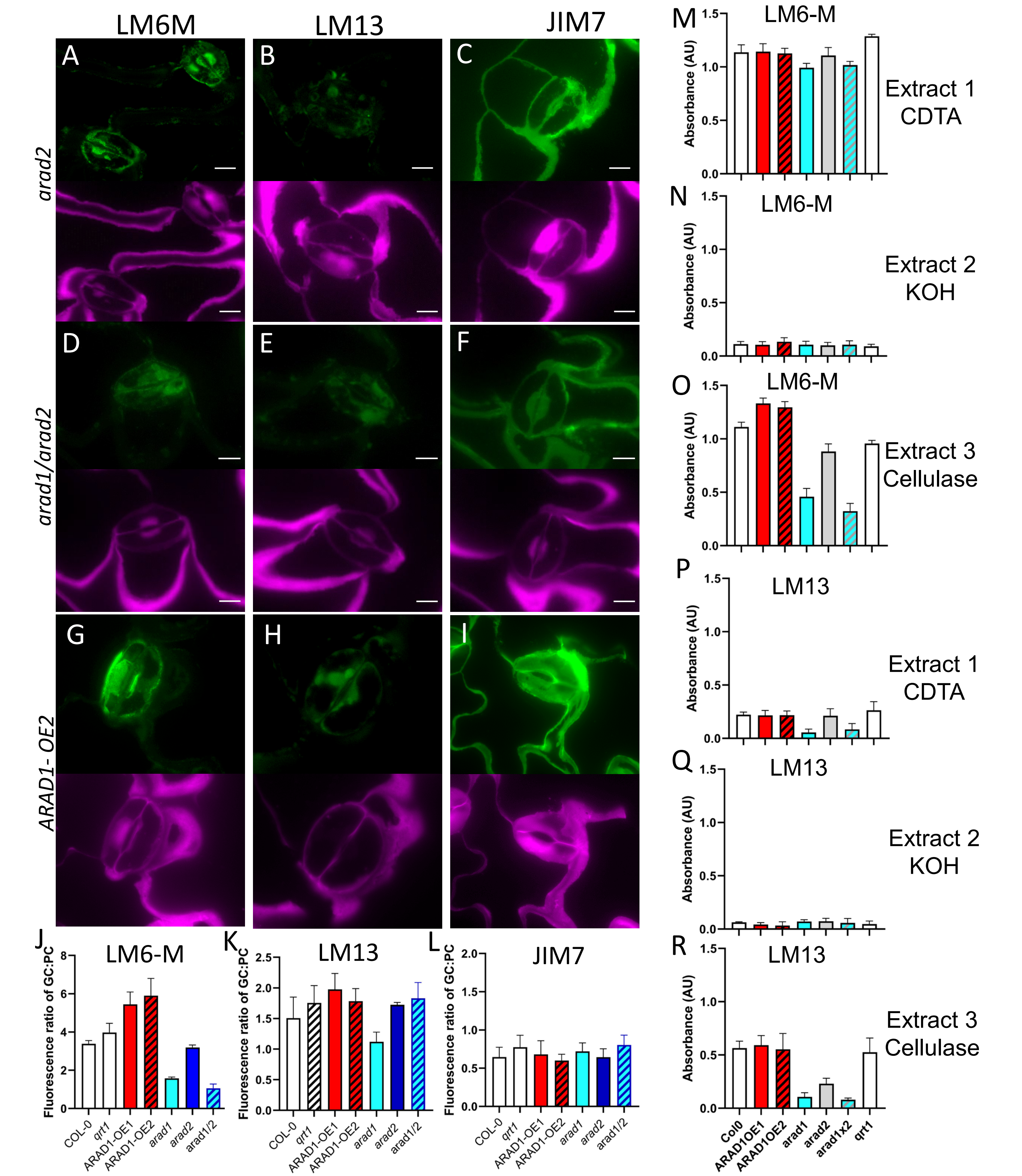

Figure S1 Antibody labelling of *arad2*, *arad1/arad2* and ARAD1-OE lines, Related to Figure 1 (A-C) Sections of *arad2* tissue incubated with antibodies against (**A**) SCL -arabinan epitopes (LM6M), (**B**) LC-arabinan epitopes (LM13) or (**C**) broad-spectrum pectin (JIM7). (**D-F**) Sections of *arad1/arad2* tissue treated as in A-C. (**G-I**) Sections of ARAD1-OE2 tissue treated as in A-C The upper panel in each figure part shows the signal (green) indicating epitope distribution. The lower panel in each figure part (purple signal) shows the general distribution of cell wall material revealed by calcofluor staining. Scale Bars = 10µm. **(J-L)** Relative fluorescence of stomatal complexes against neighbouring epidermal cells in sections. genotypes (as indicated) incubated with (**J**) LM6-M; (**K**) LM13; (**L**) JIM7. 4 biological repeats were analysed per genotype, with 3 technical replicates per sample. Error bars = SD. **(M-R)** ELISA was performed on sequential cell wall extracts (CDTA, KOH, cellulase- as indicated by extract 1, 2, 3) of rosette tissue from a range of genotypes (as indicated) probed with antibodies for **(M-O)** LM6-M (shorter chain arabinans) and **(P-R)** LM13 (longer chain arabinans). Extracts were analysed from 5 individual plants, with error bars = SD.

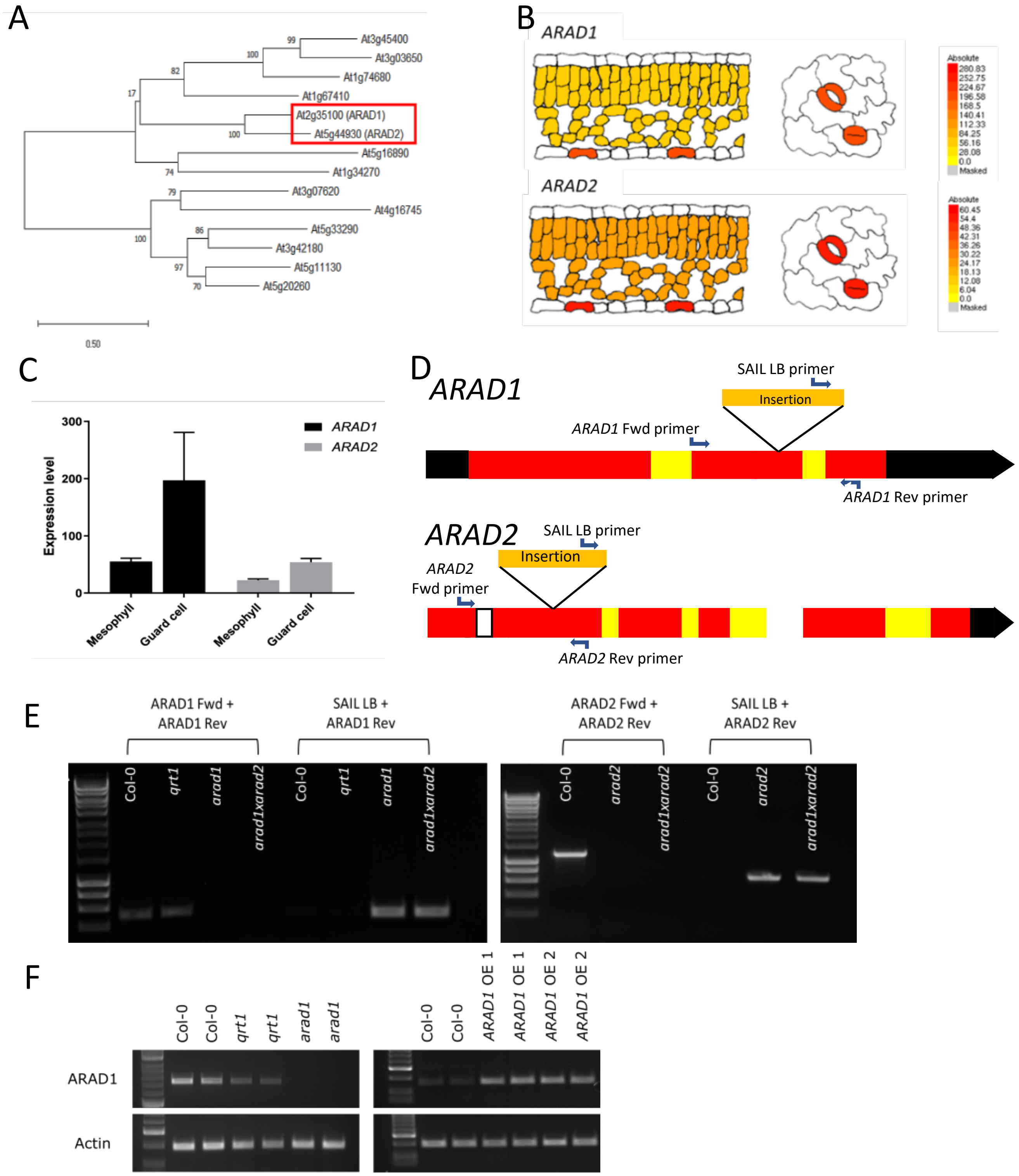

Figure S2. Arabinan genes in Arabidopsis, Related to Figure 2 (A) Identifiication of two closely related genes encoding putative arabinan synthases, *ARAD1* and *ARAD2.* Maximum likelihood tree with bootstrap testing showing *ARAD1* and *ARAD2* (highlighted in red) and the most closely related genes in *Arabidopsis thaliana.* **(B)** *ARAD1* and *ARAD2* transcripts are present in guard cells. Data from the Arabidopsis EFP browser shows the absolute expression patterns for *ARAD1* and *ARAD2* in the mesophyll and guard cell, with darker colours representing higher expression[S1]. **(C)** Quantitative comparison of the expression patterns portrayed in (B) indicates that *ARAD1* is more highly expressed in guard cells than *ARAD2*. **(D)** T-DNA mutants in *ARAD1* and *ARAD2*. Black boxes show UTRs, red shows exons, yellow shows introns and white the intergenic regions. Transposon insertion site is indicated with the genotyping primers **(E)** Identification of knock-out mutants *arad1*, *arad2* and *arad1/arad2* using primers for gene fragments specific to each genotype **(F)** Identification of ARAD1-overexpressing lines and confirmation of ARAD1 expression in the *qrt1* background. Either *ARAD1* or actin (control) gene fragments using cDNA synthesized from RNA of *Col-0*, *qrt1*, *arad1*, and independent *ARAD1* overexpression lines.

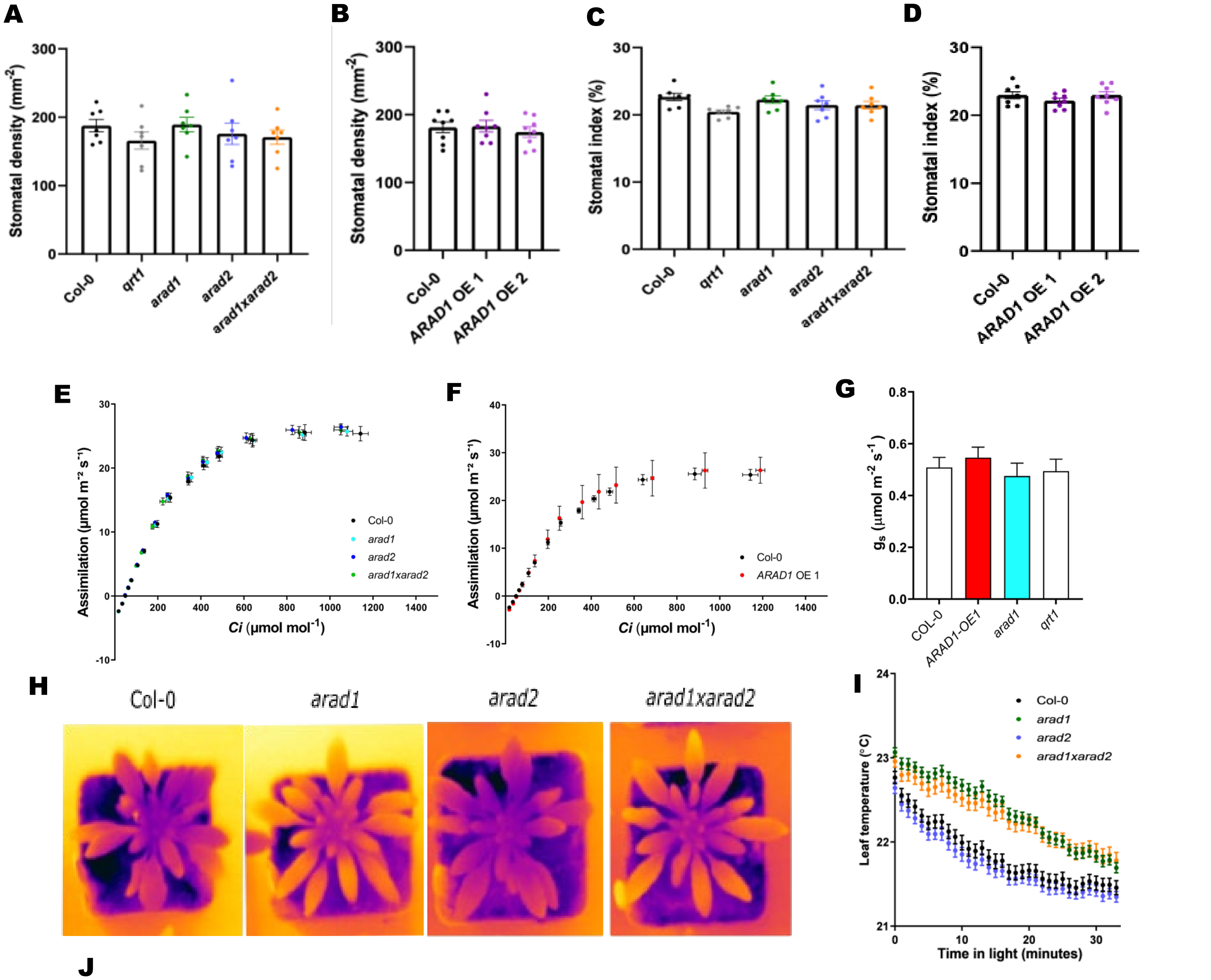

J

	arad1 vs control (qrt1)			ARAD-OE vs control (col-0)		
	Low CO_2	Ambient CO_2	Elevated CO_2	Low CO_2	Ambient CO_2	Elevated CO_2
**A**	1 ± 0.1	0.9 ± 0.1	0.85 ± 0.1	1 ± 0.1	1.1 ± 0.1	1.2 ± 0.1
**gs**	0.91 ± 0.1	0.8 ± 0.1	0.72 ± 0.1	1.1 ± 0.1	1.2 ± 0.1	1.1 ± 0.1
**iWUE**	1.1 ± 0.1	1.1 ± 0.1	1.2 ± 0.1	0.85 ± 0.1	0.9 ± 0.1	1.1 ± 0.2
**WUE**	1.1 ± 0.1	1.1 ± 0.1	1.2 ± 0.1	0.8 ± 0.1	0.9 ± 0.1	1.2 ± 0.1

Figure S3 *gs*max, stomatal patterning and underlying photosynthesis is unchanged in the arabinan mutants. Related to Figure 3. Stomatal density in (**A**) *arad1*, *arad2*, *arad1/arad2* and *qrt1* lines and (**B**) ARAD1-OE lines 1 and 2, as well as *Col-0*. Stomatal index in (**C**) *arad1*, *arad2*, *arad1/arad2* and *qrt1* lines and **(D)** ARAD1-OE lines 1 and 2, as well as *Col-0*. Each point represents a mean value calculated from a biological replicate (n ≥ 6), with columns indicating mean value and error bars = sem. Statistical analysis (ANOVA) did not support any significant variation between samples in A-D. **(E)** and **(F)** ACi curves for leaves from **(E)** *arad1*, *arad2* and *arad1/2* mutants and **(F)** ARAD1-OE transgenics did not reveal any overt change in photosynthetic performance between the mutant and control plants. Leaves from 6 independent plants were analysed to calculate the mean values shown for a range of Ci values. Error bars = SD. **(G)** *gs*max was calculated from the anatomical data shown in Table S1. ANOVA did not reveal any significant difference between the genotypes analysed (n= 8, error bars = SD). **(H)** Thermal images of *Col-O, arad1, arad2, arad1x arad2* plants after exposuere to light. Darker colours represent cooler colours. **(I)** Leaf temperature data for Arabidopsis rosettes in response to light, Error bars = SEM. **(J)** Values of assimilation rate (A), stomatal conductance (gs) and intrinsic water-use-efficiency (iWUE) and instantaneous water-use efficiency (WUE) for the mutants arad1 and ARAD-OE under low (100 ppm), ambient (400 ppm) and elevated (1000 ppm) CO2 levels relative to those for control plants grown under same conditions.

	Stoma Length (µm)	Pore Length (µm)	Aperture (µm)	GC Width (µm)
ARAD1-OE	22.470	6.250	1.040	8.210
Control	22.780	6.070	0.980	8.155
arad1	21.010	5.980	1.290	7.815

Genotype	C1 (Mpa)	C2	E (MPa)	C5 (MPa)
ARAD1-OE	1	3.33	9.99	500
Control	1	3.92	11.80	500
arad1	1	8.1	24.30	500

Table S1: FE model initial geometry and FE model parameters. Related to Figure 4
Parameters used to set the geometry of the modelled guard cells based on average measurements taken from stomata in the three genotypes analysed and Parameter fitting of the FE model that leads to model outputs capturing observed phenotypic behaviour of pore dynamics for each of the Arabidopsis genotypes described in Fig. 2. C1, C2, E and C5 are defined in STAR Methods.

Supplemental references

S1. Winter D., Vinegar B., Nahal H., Ammar R., Wilson G.V., Provart N.J. An “Electronic Fluorescent Pictograph” browser for exploring and analyzing large-scale biological data sets. PLoS One 2007;2:e718. doi:10.1371/journal.pone.0000718.
